# Phantom design and dosimetric characterization for multiple simultaneous cell irradiations with active pencil beam scanning

**DOI:** 10.1007/s00411-019-00813-1

**Published:** 2019-09-20

**Authors:** Monika Clausen, Suphalak Khachonkham, Sylvia Gruber, Peter Kuess, Rolf Seemann, Barbara Knäusl, Elisabeth Mara, Hugo Palmans, Wolfgang Dörr, Dietmar Georg

**Affiliations:** 1 Department of Radiation Oncology, Medical University of Vienna, Vienna, Austria; 2 Division of Radiation Therapy, Department of Diagnostic and Therapeutic Radiology, Faculty of Medicine Ramathibodi Hospital, Mahidol University, Bangkok, Thailand; 3 EBG MedAustron GmbH, Wiener Neustadt, Austria; 4 University of Applied Science, Wiener Neustadt, Austria; 5 National Physical Laboratory, Teddington, UK

**Keywords:** Relative biological effectiveness, Phantom, Dosimetry, EBT3 films, Proton scanning

## Abstract

A new phantom was designed for in vitro studies on cell lines in horizontal particle beams. The phantom enables simultaneous irradiation at multiple positions along the beam path. The main purpose of this study was the detailed dosimetric characterization of the phantom which consists of various heterogeneous structures. The dosimetric measurements described here were performed under non-reference conditions. The experiment involved a CT scan of the phantom, dose calculations performed with the treatment planning system (TPS) RayStation employing both the Pencil Beam (PB) and Monte Carlo (MC) algorithms, and proton beam delivery. Two treatment plans reflecting the typical target location for head and neck cancer and prostate cancer treatment were created. Absorbed dose to water and dose homogeneity were experimentally assessed within the phantom along the Bragg curve with ionization chambers (ICs) and EBT3 films. LET_d_ distributions were obtained from the TPS. Measured depth dose distributions were in good agreement with the Monte Carlo-based TPS data. Absorbed dose calculated with the PB algorithm was 4% higher than the absorbed dose measured with ICs at the deepest measurement point along the spread-out Bragg peak. Results of experiments using melanoma (SKMel) cell line are also presented. The study suggested a pronounced correlation between the relative biological effectiveness (RBE) and LET_d_, where higher LET_d_ leads to elevated cell death and cell inactivation. Obtained RBE values ranged from 1.4 to 1.8 at the survival level of 10% (RBE_10_). It is concluded that dosimetric characterization of a phantom before its use for RBE experiments is essential, since a high dosimetric accuracy contributes to reliable RBE data and allows for a clearer differentiation between physical and biological uncertainties.

## Introduction

Radiobiological research has become an integral part of particle beam therapy since its beginning (Wilson 1946). The aim of this research is twofold: first to investigate the underlying mechanisms of particle beams on biological tissue and second to compare these mechanisms with those observed in photon beam therapy. Particle beams can be several times more efficient in causing the damage in a tissue than the photon beams. The elevated radiobiological effect of different particle types is quantified by the relative biological effectiveness (RBE) (Karger and Peschke 2018). RBE depends on numerous conditions and parameters, i.e., the type of the study (in vivo, in vitro), dose, dose per fraction, cell or tissue type, biological endpoint studied, oxygen concentration, cell handling and processing, linear energy transfer (LET) and particle energy, ionization track structure on the micro- and nanoscale, etc. (Paganetti et al. 2002). In current practice, the RBE represents the basis for the estimation of the biologically weighted dose in clinical particle beam therapy (IAEA and ICRU 2008; Ödén et al. 2017; Jones 2017; Jones et al. 2018).

The vast majority of existing radiobiological data for particle beam therapy are based on in vitro studies with cultured cells irradiated in passive scattered beams. The passive scattering technique is becoming outdated nowadays and all the newly installed and planned centers for proton and ion beam therapy are at least partially, but more often entirely, based on pencil beam scanning (PTCOG 2018). Differences in RBE for scattering and scanning systems might be present due to differences in energy spectra and LET distributions, dose rates, and neutron contamination (Paganetti and Schmitz 1996). Especially the biological implications of the LET distribution in passive scattering and active scanning systems have recently stimulated research in proton therapy (Giantsoudi et al. 2016).

Radiobiological studies in particle beams are particularly challenging in horizontal beam arrangements; standard dishes for cell culturing (Kanemoto et al. 2014; Howard et al. 2018) cannot be used and also other practical issues need to be overcome. For example, cells for horizontal irradiation require sealed flasks with more nutrition medium compared to cells irradiated in vertical beam arrangements. Additionally, if the required conditions for temperature and nutrition are not entirely fulfilled, cells in horizontal configurations are more likely to disintegrate and die compared to cell in vertical configurations. So far, phantoms for in vitro cell irradiation in horizontal beams are typically designed and/or produced in-house. Typically, a block of material is added in front of the cultured cells to mimic the desired depth in tissue along the beam path (Iwata et al. 2016; Dokic et al. 2016; Howard et al. 2018). Some studies report on more advanced phantoms, although only a few provide a detailed description of these phantoms (Ando et al. 2001; Elsässer et al. 2010; Matsumoto et al. 2014).

Accurate dosimetry within a phantom at the location of the cells is essential in radiobiological research. In the existing studies, doses have been determined without incorporating all phantom materials into the treatment planning systems (TPS) or even more often, even though the phantom consisted of other materials (e.g., acrylic glass), dosimetric measurements were performed in water at a depth considering the water equivalent thickness (WET) of each non-water material. Incomplete dosimetry might lead to systematic dosimetric uncertainties in radiobiological experiments, especially in heterogeneous phantoms. Phantoms for biological experiments typically do not fulfill the dosimetric guidelines established for reference dosimetry (Andreo et al. 2006). Consequently, direct extrapolation of the results from the literature for reference dosimetry is not feasible.

The aim of this study was to establish a systematic and comprehensive dosimetric characterization of an in-house phantom developed for in vitro studies in horizontal particle beams, which enables simultaneous irradiation at multiple positions along the beam path. Dosimetry was performed under non-reference conditions, since the material composition and geometry of the phantom did not fulfill the requirements for reference dosimetry stipulated in IAEA TRS-398 (Andreo et al. 2006).To obtain exact dose-averaged LET values (LET_d_) for each cell position, LET values were calculated in research TPS RayStation (RaySearch Laboratories, Sweden, V5.99) using an existing Monte Carlo (MC) dose algorithm (RaySearch Americas Inc. 2017; Saini et al. 2018). The LET information obtained directly from the TPS system allows common users to estimate experimental or model-based RBE values. Finally, the phantom was used for irradiation of a melanoma (SKMel) cell line including a complete dosimetric characterization of the experiment.

## Materials and methods

### Phantom for biological cell irradiation

**Fig. 1:**
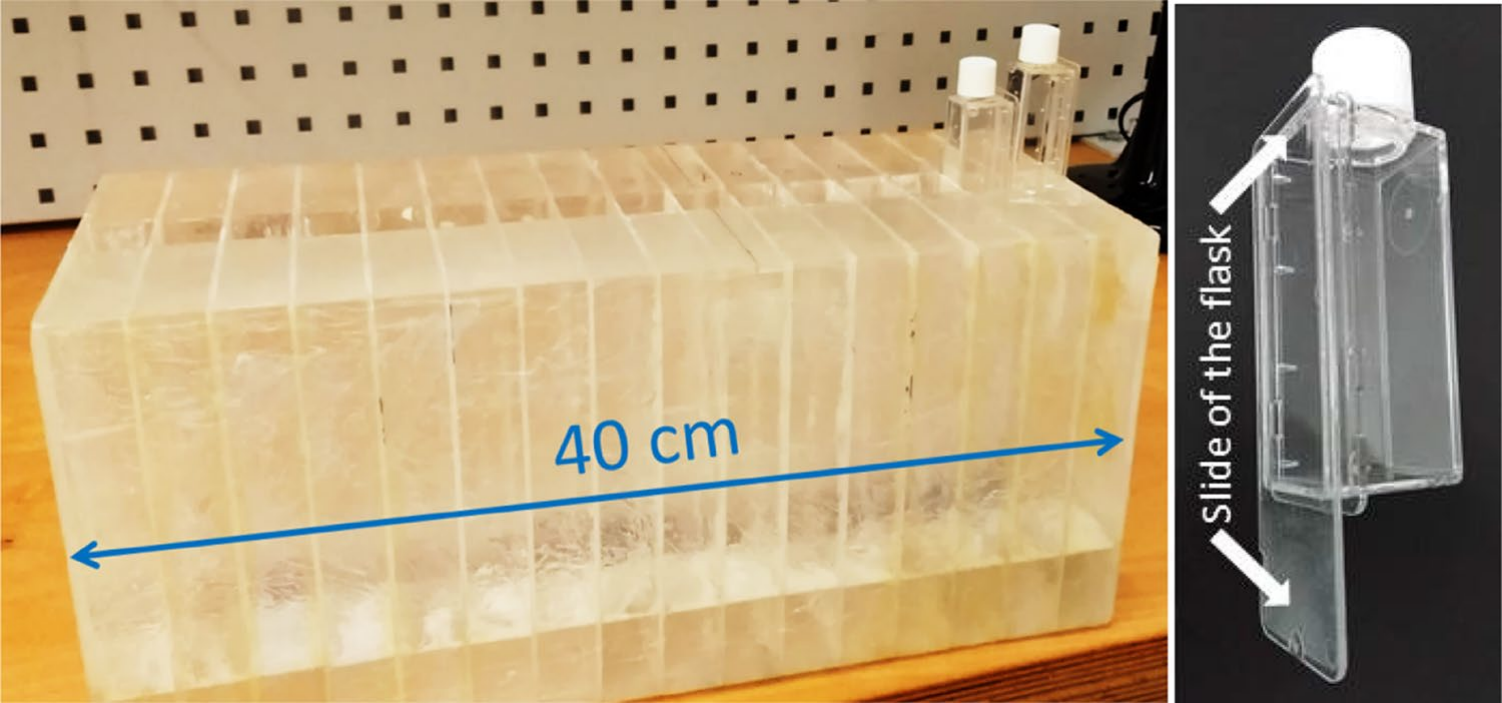
Left: heterogeneous phantom for in vitro studies with two plastic flasks for cell cultivation and irradiation. The phantom has 16 inserts for cell flasks over a length of 40 cm. Right: plastic flask for cell studies. Cells are cultured at the inner side of the slide

The phantom shown in Fig. 1 was developed for radiobiological studies in a dedicated research room at MedAustron, the synchrotron-based Austrian center for ion beam research and cancer treatment (Stock et al. 2017). This research room is equipped with a horizontal beam line including a quasi-discrete spot scanning technique with active energy variation for proton and carbon ions commissioned according to clinical specifications. The maximal field size is 20 × 20 cm^2^ and the spot size of the beam varies from 4 to 10 mm FWHM (full width half maximum), depending on the beam energy.

To facilitate positioning of biological samples, the phantom consists of acrylic glass (PMMA) with 16 inserts, which are separated by PMMA plates of 2 mm in thickness. These inserts are 23 × 25 × 100 mm^3^ (*L* × *W* × *H*) in size, to house specific air-tight flasks (Thermo Scientific, Nunc, Penfield, NY, USA). Cells were cultured in a monolayer on a slide from the inner side of the flask (Fig. 1, right). The advantage to seed the cells in flasks to be inserted in the phantom instead of seeding the cells directly in the phantom is the decreased chance for cell contamination. Moreover, this configuration allows quicker cell flask exchange and thus more efficient irradiations, since there is no need to clean the phantom between irradiation sessions. To sustain the dosimetric homogeneity of the phantom and to avoid possible air in the setup, the area around the flasks in the phantom is filled with about 5 ml of distilled water. The flask itself is filled with a medium (e.g., MEM—minimum essential medium) to supply the cells with basic nutrients.

The maximum phantom length of 400 mm allows measurements along the entire energy range of clinical relevance. For example, the Bragg peak of the highest proton energy (252.7 MeV) clinically utilized at MedAustron terminates in the 15^th^ compartment of the phantom (i.e., at 380 mm in water). The phantom can be irradiated with single fields as well as two opposing irradiation fields to simulate patient treatment configuration.

### Water equivalent thickness (WET) of phantom materials

To ensure that the phantom materials were assigned to correct Hounsfield units (HU) and thus relative stopping powers for subsequent dose calculation in the TPS, WETs of the phantom, flask and various media for cell supply were determined. The term WET in g/cm^2^ refers to the product of the actual thickness (in cm) and the material mass density (in g/cm^3^) (Andreo et al. 2006).

**Fig. 2:**
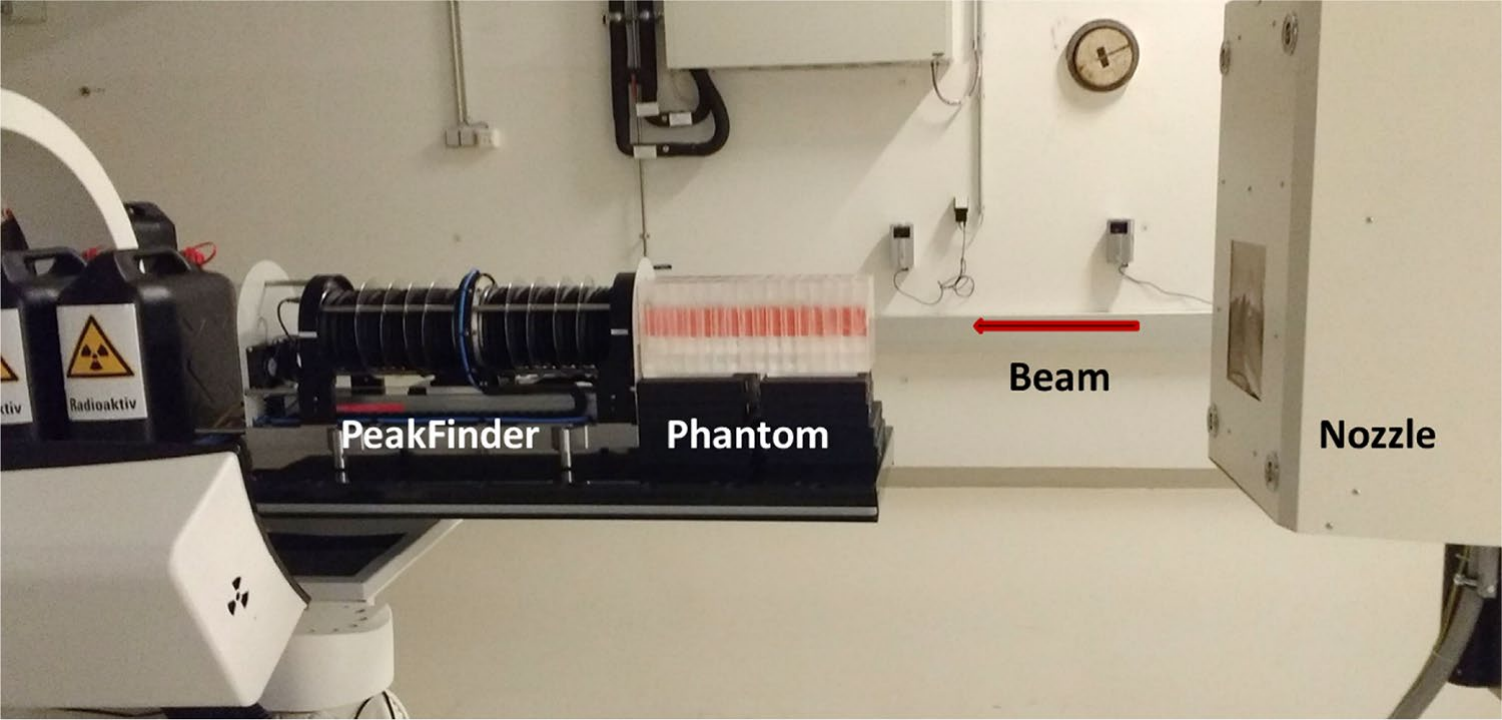
Phantom setup for WET measurements with PeakFinder; flasks with cell medium (MEM) are inserted in the phantom compartments. The horizontal beam exits through the nozzle on the right

More specifically, WETs of the phantom components were derived experimentally at a proton beam energy of 198 MeV using a comparative range measurement method. Proton beam ranges were measured with the PeakFinder (PKF) water column system (PTW, Freiburg, Germany), shown in Fig. 2. The phantom was prepared for irradiation, consisting mainly of cell medium, PMMA and plastic mini-flasks. The empty phantom, the phantom with materials of interest, or a stack of several samples was placed in front of the PKF. The depth dose curve was recorded in steps of 0.1–0.5 mm with the parallel-plate ionization chamber (Bragg peak, type 34080, PTW, Freiburg, Germany) of the PKF. The proton beam ranges (*R*_80_) were evaluated with the PeakScan software (PTW, Freiburg) at 80% of the distal end region of the recorded Bragg peak. The WET was defined as the difference of the measured ranges without any material and the material of interest.

WETs of phantom materials were taken into account for plotting the depth dose distribution obtained from film and IC measurements as well as TPS data, as presented in the results section below.

### Proton irradiation and measurement setup

#### Plan design

The workflow for the dose assessment and later for the dose delivery to the cultured cells was similar to that of a real patient treatment. A CT scan of the phantom was acquired with a Philips Brilliance-CT Big Bore scanner system (Philips, Eindhoven, The Netherlands). The CT scan included 16 flasks filled with distilled water; each compartment around the flask was also filled with distilled water.

**Fig. 3:**
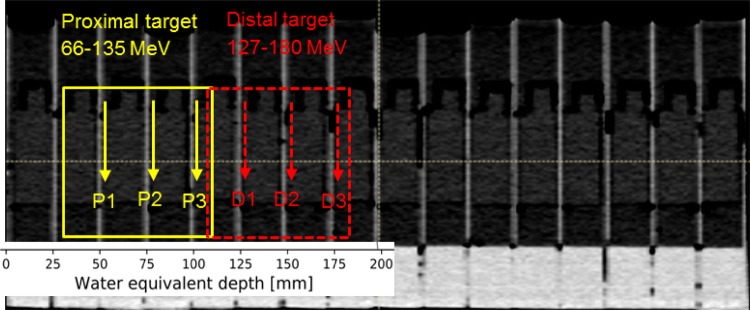
CT scan of the phantom in TPS with two illustrated targets. Cell positions are indicated with solid vertical yellow lines for the proximal (positions P1, P2, and P3) and dashed vertical red lines for the distal target (positions D1, D2, and D3). EBT3 films were located for the depth dose measurements at the same positions as cells (color figure online)

Treatment plans were created and dose calculations were performed utilizing the RaySearch TPS RayStation. Two cell irradiation geometries were chosen to reflect a target location at a proximal depth (as, e.g., head and neck cancer) and at a distal depth (as, e.g., prostate tumors). Geometries defined (based on the CT scan of the phantom) covered three compartments of the phantom to represent RBE values at three different positions within the spread-out Bragg peak (SOBP). Figure 3 illustrates these scenarios, i.e., a box-shaped target with dimensions of 80 × 60 × 80 mm^3^ (*L* × *W* × *H*) for the proximal and a second one of 70 × 60 × 80 mm^3^ for the distal target. These setups required proton energies in the range between 66.5 and 135.6 MeV (range 115 mm, modulation 80 mm) for the proximal target and between 127.2 and 180.1 MeV (range 180 mm, modulation 70 mm) for the distal target. The physical doses in the center of the SOBP were set to 2 Gy for both scenarios. Two separate treatment plans with a single-field irradiation were generated in TPS employing either the Pencil Beam (PB) or the MC dose algorithm (RaySearch Americas Inc. 2017; Saini et al. 2018). LET_d_ values were calculated using the MC dose engine available in the research version of RayStation.

The phantom was placed on the robotic couch in the research irradiation room and the isocenter position defined in the TPS was aligned using the room lasers. The isocenter for the proximal target was defined and aligned at the phantom entrance surface. The distal target was aligned in the middle of the SOBP. Irradiation times were between 15 and 20 min for both targets. The dosimetric measurements were carried out with Pinpoint ionization chambers (PinPoint IC, Type 31015—0.03 cm^3^, PTW, Freiburg, Germany) and EBT3 Gafchromic films (International Specialty Product, NJ, USA), which are described in more detail in the following subsections.

#### Ionization chambers

Air-vented, small volume (“PinPoint”) cylindrical ionization chambers were placed along the central beam axis in every insert of the phantom to cover the entire irradiated region in depth. To obtain a stable response of the PinPoint IC, pre-irradiation of 15 Gy was delivered before each measurement (Carlino et al. 2018). One IC per compartment was positioned as close as possible to the front wall of the insert (towards the nozzle). This means that, due to the chamber size (diameter: 7 mm), the effective positions for the ionization chambers were not identical to the cell positions. The effective point of measurement was taken as a measurement point 0.75 × *r*_cyl_ closer to the phantom surface than the center of the IC, where *r*_cyl_ is the inner radius of the chamber (Palmans 2006). Two ICs were used for the measurements at the same time, with their sensitive volumes shifted in height to avoid shielding effects. All IC were connected to the electrometer MULTIDOS (type 10004, PTW, Freiburg) to measure the charge in electrical units. The temperature and pressure correction factor (*k*_TP_) as well as individual chamber calibration factors (*N*_D,W_) were applied. For each Pinpoint IC, a specific calibration factor was acquired beforehand by cross-calibration against a reference Farmer IC, under reference conditions in proton beams according to the TRS398 protocol (Andreo et al. 2006; Carlino et al. 2018). The reference Farmer IC is regularly sent to an accredited standards laboratory, where calibration of the absorbed dose to water in a ^60^Co beam is performed. Absorbed dose to water in the proton beams using the Farmer chamber was obtained as the product of the IC reading corrected for influence quantities, the absorbed dose to water calibration coefficient and a beam quality correction factor (*k*_Q_) for the Farmer IC of *k*_Q_ = 1.029 (Andreo et al. 2006).

The reference Farmer IC (Type 30013—0.6 cm^3^, PTW, Freiburg, Germany) was additionally employed for the dose determination within the SOBP. Similar to the PinPoint IC procedure, an effective point of measurement setup correction (Farmer IC diameter: 12.6 mm) was performed.

#### Gafchromic films

Depth dose measurements were performed using EBT3-type Gafchromic films, an ideal small-size and flexible dosimeter of which multiple samples can be placed simultaneously at several depths allowing discrete multidimensional dose assessment in a one-shot irradiation. On the other hand, the energy dependence of these films and their under-response in the Bragg peak are well-known disadvantages (Zhao and Das 2010; Kirby et al. 2010; Reinhardt et al. 2012). Films were cut into pieces of 25 × 60 mm^2^ and positioned perpendicular to the beam on the same side of flask (see Fig. 1, right), where cells would be platted. The slides were separated from the rest of the flask for this purpose and the films were attached to the slide from the inner side of the flask. The film and cell positions are also indicated in Fig. 3. Flasks with films were placed into the respective phantom inserts and the rest of the phantom was filled with distilled water.

Film handling was performed according to the AAPM TG-55 report (Niroomand-Rad et al. 1998). Signal readout of each film was performed using an EPSON 11000 XL flatbed scanner (Seiko EPSON Corporation, Nagano, Japan) according to the procedures as outlined in Dreindl et al. (2014). Prior to irradiation, a background signal was obtained. Irradiated films were scanned and digitized 24 h (< 48 h) after the irradiation. Film orientation during scanning was always identical for the background and post-irradiation readings. The central area of 10 × 10 mm^2^ and 15 × 40 mm^2^ of each film was evaluated for the determination of absolute dose and dose homogeneity within the target at each depth. For the film analysis, the red channel was considered and pixel values (PVs) were analyzed using the IMAGEJ v2.0 software (National Institute of Health, USA). Three scans were acquired for each film and the mean PV with the corresponding standard deviation in the region of interest (ROI) was calculated. The PVs were converted into net optical densities (netOD) by subtracting the film background values (Devic et al. 2005; Dreindl et al. 2014). More details can be found also in a recently published study on proton dosimetry with EBT3 and EBT-XD films (Khachonkham et al. 2018). Cross-calibration of representative samples of the same film batch was performed indirectly against the Pinpoint IC chamber. The PinPoint IC measurements agreed well with TPS data (MC based) and since the film positions were shifted relative to the PinPoint chamber positions, the TPS data were used to convert the film response (net OD) to the radiation dose for absolute dose determination. Only data points shallower than the SOBP, where film exhibits no under-response, were taken for the cross-calibration. The first two and five measurement points were considered for the cross-calibration in the proximal and distal target, respectively. A constant uncertainty of 3% on the TPS dose, obtained from commissioning of the MC algorithm-based TPS, was taken into account for the final film uncertainty estimation.

### Cell line experiments

To exemplify the phantom application for radiobiological experiments in scanned proton beams, an in vitro model with a high (*α*/*β*)_*x*_ value, i.e., melanoma cell (SKMel) lines, was selected. SKMel were maintained in MEM (minimum essential medium Eagle Gibco), supplemented with 10% fetal calve serum, 5% HEPES, 2 nM l-glutamine and 1% penicillin and streptomycin.

All cells were cultured at 37 °C in a humidified atmosphere with 95% air and 5% CO_2_. Cells were seeded in chamber flasks with plastic slides, shown in Fig. 1 (right), at 2.5–5 × 10^5^ cells per flask 24–48 h before irradiation to achieve 70–80% confluence at the time of irradiation. Immediately prior to irradiation, the chamber slide flasks were filled with the respective supplemented medium.

The reference beam for cell irradiation was a photon beam with peak energy of 200 kV and the following filtration: 3 mm Be + 3 mm Al + 0.5 mm Cu. For the protons, the results are presented for the positions D1, P2, P3 (Fig. 3) for the proximal and distal target, respectively. Each experiment was repeated at least three times.

Standard clonogenic survival assays were performed after reference X-ray or proton irradiation. Cells were harvested immediately after irradiation with 0.05% trypsin–EDTA (Gibco) and incubated for 5–8 min at 37 °C in 5% CO_2_. Cells were diluted with supplemented medium appropriate for the cell line and seeded on 6-well dishes in concentrations according the dose level of 250 cells (0 Gy and 0.5 Gy), 500 cells (1 Gy, 2 Gy), 1000 cells (4 Gy) and 2000 cells (6 Gy) per well. Following a cell line-specific incubation period, colonies were fixed with 96% methanol and stained with 0.5% crystal violet solution. A minimum of 50 cells were considered as a colony.

Based on a linear quadratic (LQ) model, surviving fractions in reference to the plating efficiency of non-irradiated control samples were calculated for each delivered physical dose (in Gy). A 1/*σ*-weighted minimum Chi square estimation was applied to the linear quadratic model for survival curve fitting and Python 3.6 programming language (Python Software Foundation) was used for statistical procedures and graphical illustrations. The parameters *α* and *β* of the LQ model were calculated for both radiation types using the same fitting method. Furthermore, RBE values were extracted from the obtained cell survival curves by comparing the doses of X-rays with those of protons at the same level of survival (Paganetti 2014).

## Results

### WET of phantom materials

Results of WET and of the water equivalent ratio WER (ratio of WET and physical thickness) of plastic flasks, PMMA, EBT3 film, and three different cell media are summarized in Table 1. The given values result from three measurements. Obtained statistical uncertainties of three measurements were all below 2%.

**Table 1:** Values of water equivalent thicknesses (WETs) and water equivalent ratios (WERs) of materials used in the phantom

Material	Physical thickness (mm)	WET (mm)	WER
Flasks (empty)	1.4	1.4	1.00
PMMA plates	3.2	3.6	1.13
EBT3 film	0.3	0.4	1.33
Medium DMEM	20	20	1.00
Medium RPMI	20	19.9	1.00
Medium MEM	20	20	1.00

### Dosimetric characterization

**Fig. 4:**
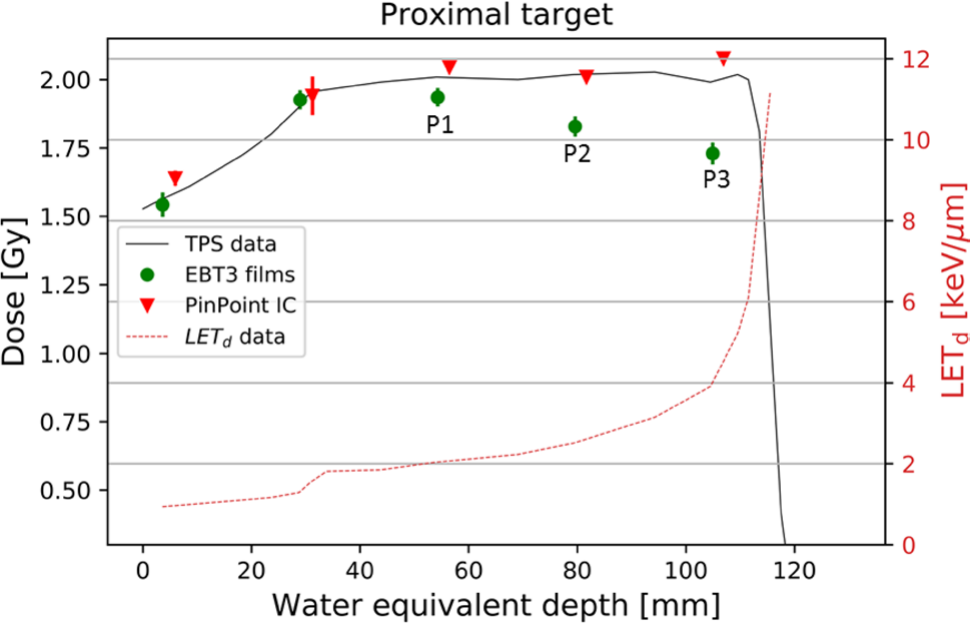
Central axis depth dose distribution obtained with EBT3 films and PinPoint IC for the proximal target. The doses were calculated in TPS employing the MC algorithm. Films were at positions P1, P2, and P3 (Fig. 3), where also cells would be plated. The solid line represents the TPS data. The values shown are the average of the results of three independent measurements. Error bars represent the corresponding standard deviations. The dose-averaged LET_d_ depth profile obtained from TPS (RayStation, V5.99) is also shown (dashed line and right *y* axis)

**Fig. 5:**
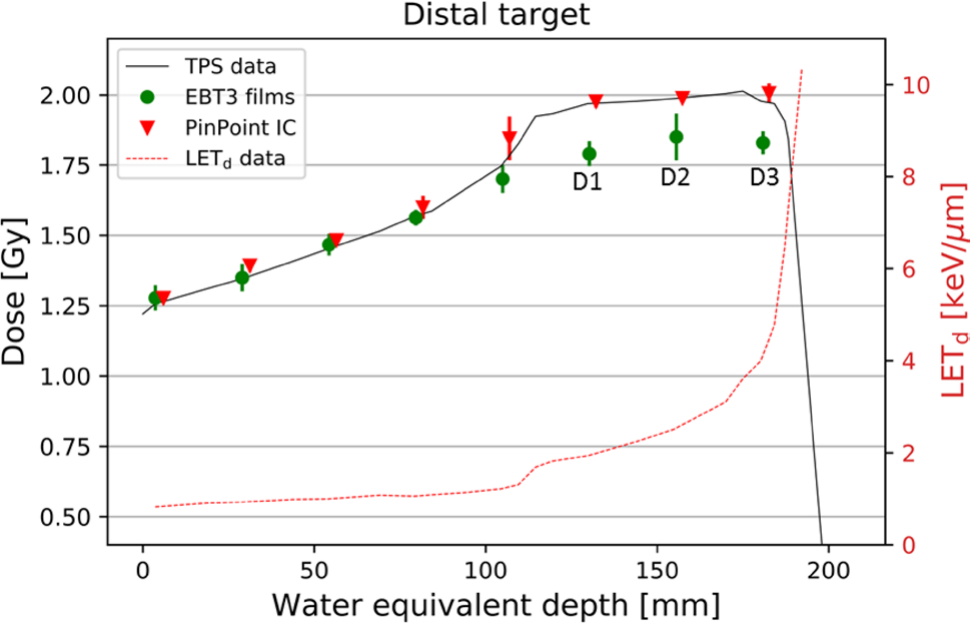
Central axis depth dose distribution obtained with EBT3 films and PinPoint IC for the distal target. The doses were calculated in TPS employing the MC algorithm. Films were at positions D1, D2, and D3 (Fig. 3), where cells would be plated. The solid line represents the TPS data. The final values shown are the average of the results of three independent measurements. Error bars represent the corresponding standard deviations. The dose-averaged LET_d_ depth profile obtained from TPS (RayStation, V5.99) is also shown (dashed line and right *y* axis)

Absorbed dose to water in the proximal target region determined by the PinPoint ICs was in good agreement with the TPS data for both MC and PB algorithms. The largest difference of 3% was obtained at the last measurement point of the SOBP in the proximal target. For the distal target, the TPS data, based on the MC algorithm, agreed well with those obtained with the ICs. The TPS data based on the PB algorithm display also sufficient target coverage in TPS. However, doses measured with the PinPoint ICs were up to 4% smaller at the last measurements point of SOBP than those calculated by the TPS. Measurements with the Farmer IC confirmed the Pinpoint IC results, and the values agreed within 1% in the investigated region. Depth dose distributions obtained with EBT3 films, PinPoint IC and TPS are compared in Fig. 4 for the proximal and Fig. 5 for the distal target. Measured values for PinPoint ICs are averaged values of three independent measurements. The averaged value of the three independent measurements performed with EBT3 films was indirectly cross-calibrated with the PinPoint IC as described in the Gafchromic films section. Propagation of uncertainties, taking into account three film measurements, the TPS uncertainty and the uncertainty associated with the cross-calibrated factor, were considered for the final film uncertainty.

For the EBT3 film measurement, the standard deviations were less than 2% within the central area of 1 × 1 cm^2^. Considering the uniformity of the film itself, variations of up to 1% obtained from background measurements for non-irradiated films were considered reasonable. For the proximal target (Fig. 4), the film underestimated the dose by up to 13%. For the distal target, EBT3 doses were smaller by 6–8% (Fig. 5). In contrast, in the steep dose gradient before the SOBP, the EBT3 film doses agreed well with the TPS data. At the last measurement point before the SOBP, the final film uncertainty is a factor of two smaller compared to that of the cylindrical IC. This is attributed to the positioning uncertainty of the Pinpoint IC and a better reproducibility of the positions of the EBT3 films which were placed always against the slide window of the flask. To characterize the dose variation (homogeneity) in lateral direction at each depth along the beam, the 15 × 40 mm^2^ area of each film was analyzed. Dose homogeneity was within 3% (1 sigma) for all irradiated films. For comparison, the dose homogeneity of the same area obtained from TPS calculations was within 1%.

**Fig. 6:**
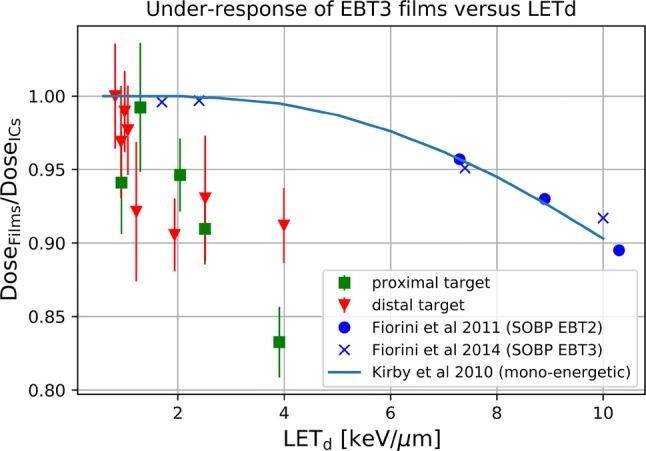
Film under-response as a function of LET_d_ for both targets, as compared to data obtained from the literature. *SOBP* spread-out Bragg peak

Calculated LET_d_ values (in keV/µm) were 2.1 (0.1), 2.8 (0.1), and 4.5 (0.3) for the measurement positions P1, P2, and P3 of the proximal target and positions D1, D2, and D3: 1.9 (0.1), 2.5 (0.1), and 4.1 (0.3) of the distal target, respectively. The film response as a function of LET_d_ for both targets is shown in Fig. 6.

### Cell line experiments

**Fig. 7:**
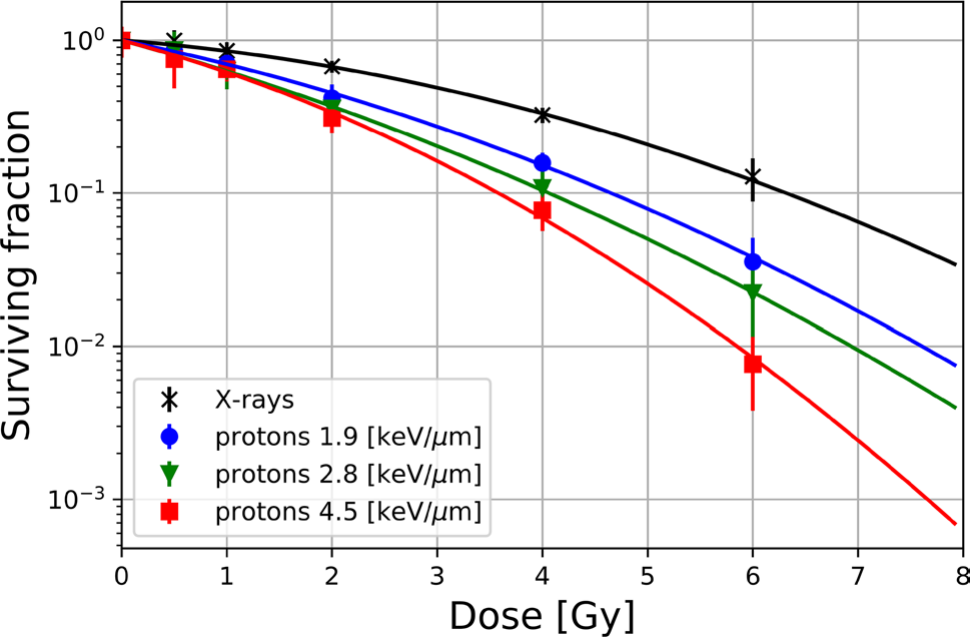
Survival curves of SKMel cells irradiated with 200 kV X-ray beam (peak energy) and proton beams at three different depths in the phantom (D1, P2, P3)

Survival curves of SKMel cell line as a function of dose and LET_d_ are illustrated in Fig. 7. The evaluated (*α*/*β*)_*x*_ for X-rays was 3.2 ± 0.7 Gy. The *α*/*β* values for protons varied from 8.7 ± 1.8 Gy (D1) to 6.6 ± 1.5 Gy (P3). RBE_10_ value (which is the RBE for a surviving fraction of 10%) was 1.4 ± 0.3 at position D1 and increased to 1.8 ± 0.2 at the last investigated depth P3.

## Discussion

In this work, a new phantom for simultaneous cell irradiation at multiple positions in horizontal particle beams is presented. The phantom is especially practical for synchrotron-based facilities, where due to the spill structure of the beam and lower dose rates, the dose delivery is substantially slower compared to continuous beams from cyclotron-based facilities. Placing multiple cell monolayers simultaneously at different positions for a single irradiation is an effective approach to perform comprehensive measurements and save research beam time. The present phantom is particularly suitable for experimental RBE determination and investigation of any RBE variations within the SOBP. Considering the inhomogeneous construction of the phantom with numerous interfaces of water (cell medium) and PMMA, in- and out- scatter from the surrounding PMMA and variable flask thicknesses, measurements in the distal fall-off of the Bragg peak, are not feasible. Additionally, elementary composition of phantom materials which are frequently in use, i.e., PMMA, introduces range changes which originate from non-elastic nuclear interaction cross-sections (Palmans et al. 2002; Lourenço et al. 2017). The preferred phantom for measurements in the distal fall-off would be a homogeneous water phantom, where two or three flasks with cultured cells would be placed behind each other and irradiated simultaneously.

Determination of the dose distribution within a phantom used for cell irradiations is essential to correctly estimate the dosimetric uncertainty involved in cell experiments. The present phantom is a heterogeneous phantom and the dose measurements were performed under non-reference conditions, for exactly the radiation field to which the cells were exposed. High dosimetric accuracy contributes to reliable RBE data and allows for a clearer differentiation between physical and biological contributions to RBE uncertainty. The procedure applied here using of a CT scan of the phantom, employing TPS for the correct material assignment to CT numbers and performing dosimetric verification directly within the phantom shall be performed before the phantom is used for cell irradiation. For the presented phantom, the depth dose measurements were performed with different types of detectors: Farmer IC, PinPoint IC, and EBT3 Gafchromic films. These detectors complement each other and provide sufficient data on the absolute dose at each cell position, as well as information on the dose distribution within each flask containing cultured cells. Plane-parallel ICs are beneficial and recommended for the depth dose measurements in steep gradients of proton beams (Andreo et al. 2006). However, limitations of using the plane-parallel IC for the measurements were the small compartment size of the phantom (i.e., 23 mm) and the lack of suitable waterproof plane parallel ICs. Other detectors suitable for high-spatial resolution measurements without the under-response observed for the EBT3 films are silicon-based detectors (Anderson et al. 2017; Tran et al. 2017). Even though the Farmer IC (volume 0.6 cm^3^) is considered as a standard detector used for absolute dosimetry in proton beams, the PinPoint IC was preferred for depth dose measurements along the modulated beam here, due to its smaller sensitive volume (0.03 cm^3^) and relevant use in the depth dose gradients. The good agreement between the Farmer IC and PinPoint IC within the SOBP (the region of uniform dose) confirmed sufficient pre-irradiation and appropriate use of PinPoint IC chambers for depth dose measurements.

Dose calculations based on the PB algorithm underestimated the dose in the developed phantom. The doses calculated with the TPS and measured with the applied dosimeters differed by 4% utilizing PB calculations at the last measurement point (D3) of the distal target. The difference between the calculated and measured doses is larger for the distal target, which is deeper in the phantom and thus the beam passes, due to the inhomogeneous construction of the phantom, more interfaces between cell media, flasks and phantom PMMA walls. Similar observations of reduced dose using the PB algorithm for heterogeneous phantoms are reported in Saini et al. (2017, 2018), where doses from two algorithms (PB, MC) of the commercial RaySearch TPS were compared for inverse plan optimization and final dose calculations with doses obtained from phantom measurements. In the present work, the MC calculations agreed with the measured doses in the phantom. This underlines the importance of using an MC-based algorithm to deal adequately with the issue of material heterogeneity.

All cells cultured in one flask were considered and processed for the evaluation of RBE and only one RBE value was extracted, which means that also the homogeneity of the dose within the flask was an important parameter. EBT3 films are one of very few detectors applicable to perform high-spatial resolution measurements in lateral dose assessment. This is also true for the region of the Bragg peak, where films undergo quenching, under the assumption that the quenching is uniform within one layer. Dose homogeneity was within 3% for all irradiated films, including the regions with steep dose gradients. For absolute dose determination, the films were cross-calibrated against the PinPoint IC. The data points before the SOBP, where the films experience no quenching, were used for cross-calibration. The films demonstrated a more accurate outcome at steep gradient regions of the SOBP curve than the cylindrical ICs used, which is attributed to their small thickness and reproducible positioning. Under-response in the region of SOBP confirmed the expected film saturation in high-LET regions. As a consequence, the measured EBT3 dose values were 6–13% lower in the SOBP than those measured with the ICs or calculated with the TPS. The EBT3 under-response in the SOBP was higher for the proximal target (with lower applied energies) than for the distal target which is in agreement with other studies on film energy dependence (Zhao and Das 2010; Fiorini et al. 2014; Khachonkham et al. 2018). In the present study, the film response was evaluated as a function of calculated LET_d_ obtained from the already existing MC dose framework in the TPS. The obtained under-response values derived from the LET_d_ values for both targets were lower compared to the data published for mono-energetic protons (Kirby et al. 2010) and SOBP data with EBT2 films (Fiorini et al. 2011) and EBT3 films (Fiorini et al. 2014) (Fig. 6). Among possible reasons are larger uncertainties obtained for films in this work, phantom inhomogeneity, scanner or scanning protocol, small film sizes or recently discussed changes in EBT3 film production (Prof. Larry DeWerd, personal communication, 2018).

The cell survival curves of SKMel cells, irradiated with proton beams simultaneously at three positions with different LET_d_, were progressively steeper than those of cells irradiated with X-rays, at all investigated positions, indicating a higher number of cell deaths and cell inactivations after high-LET exposure even though receiving the same physical dose. The observed increase in RBE corresponds to an increase in LET_d_, which indicates that RBE is dependent on LET_d_ for this investigated cell line. It is noted that RBE values substantially higher than the clinically used value of 1.1 were obtained for all three positions. More specifically, RBE values of 1.4 and 1.8 were obtained for RBE_10_. This result suggests that SKMel cells show a much better response to proton beams compared to X-ray beams than an RBE value of 1.1 would imply.

In the present study, the focus was to obtain the doses and LET_d_ values in the investigated phantom at the exact positions of biological samples leading to an appropriate basis for the RBE determination from the experiments as well as for RBE models. Existing RBE models are based on proton dose, dose-averaged LET_d_ and tissue-specific parameters of the linear quadratic (LQ) model (Carabe et al. 2012; Wedenberg et al. 2013; McNamara et al. 2015). Underestimation of proton dose in TPS might lead to an overestimation of the corresponding RBE value. For example, the observed underestimation of dose from the PB algorithm by 4%, resulted in a 1.5% higher RBE value at the last measurement point of the distal target (D3 in Fig. 5). These results are based on the analytical expression of the RBE (Wilkens and Oelfke 2004) with input from SKMel cell irradiations performed in the presented phantom.

## Conclusion

In the present study, a novel phantom was constructed for in vitro cell irradiations and dose verification measurements. The phantom enables simultaneous cell irradiation at up to 16 positions along the Bragg curve for a horizontal research ion beam line with active scanning technology. The highly accurate dose measurements performed in the phantom contribute to reliable RBE data and allow for a clearer differentiation between physically motivated and biologically motivated uncertainties. Dose verification was performed with EBT3 films and cylindrical ionization chambers. The use of different systems for dose measurements facilitated the identification of possible systematic errors. MC-based treatment plan calculations are essential for the biological studies to overcome the issue of heterogeneity in inhomogeneous phantoms. To exemplify the phantom application for radiobiological experiments in scanned proton beams, an in vitro model with a high (*α*/*β*)_*x*_ value, i.e., melanoma cell (SKMel) line, was applied. The obtained RBE values are substantially higher than the clinically used value of 1.1 and their LET_d_ dependence is apparent. This is an important finding not only for the possible overdose of the tumor but also for the healthy neighboring tissue being exposed to radiation with higher than expected RBE values leading to more biological damage. It is concluded that the presented phantom is a valuable tool for biological studies of RBE values in particle beams. Different light ion species in active scanning configuration are foreseen for future in vitro studies at the MedAustron center. In these studies, the phantom described in the present paper and improved designs based on this study will be used.
